# Record of the invasive alien ladybird *Harmonia axyridis* (Coleoptera, Coccinellidae) from Kenya

**DOI:** 10.3897/zookeys.106.1242

**Published:** 2011-06-15

**Authors:** Oldřich Nedvěd, Jiří Háva, Daniela Kulíková

**Affiliations:** 1 Faculty of Sciences, University of South Bohemia, and Institute of Entomology, Biology Center, Academy of Sciences of the Czech Republic, Branišovská 31, 370 05 České Budějovice, Czech Republic; 2 Private Entomological Laboratory & Collection, Rýznerova 37, CZ-252 62 Únětice u Prahy, Praha-západ, Czech Republic; 3 Podřipská 188, CZ-41185 Horní Beřkovice, Czech Republic

**Keywords:** Multicolored Asian Ladybird, distribution, new record, Kenya, Afrotropical region, invasive predator

## Abstract

The biological control agent and alien invasive ladybird *Harmonia axyridis* (Pallas, 1773) was recorded for the first time in Kenya, and in equatorial Africa, in 2010.

## Introduction

The multicolored Asian lady beetle or harlequin ladybird *Harmonia axyridis* (Pallas) (Coleoptera: Coccinellidae) is native to temperate (and mountain subtropical) Central and East Asia: China, Taiwan, Japan, Korea, Mongolia, Kazakhstan and eastern Russia (Kuznetsov 1992). It was introduced in many regions of the world as a biological control agent against aphids, and later became an invasive species, spreading 100–500 km each year. It is established in at least 37 countries in four continents (Brown et al. in press). In Africa, this species was intentionally introduced in two Mediterranean countries: Tunisia, where it did not survive, and Egypt (Ferran et al. 2000), where it established a limited population (Brown et al. in press). Conversely, it has invaded and established in South Africa (Stals and Prinsloo 2007) and neighbouring Lesotho (Stals 2010) although it was not intentionally introduced there.

This article reports the first record of this alien invasive ladybird beetle in Kenya.

## Material examined

Kenya E, Coast province, Kikambala (3°48.28'S, 39°50.00'E; cca. 45 km N of Mombasa), 30.12.2010–8.1.2011, 2 ♀♀ lgt. + 20 exx. observ., Jiří Háva & Daniela Kulíková lgt., J. Háva coll. et det.

**Figure 1.**
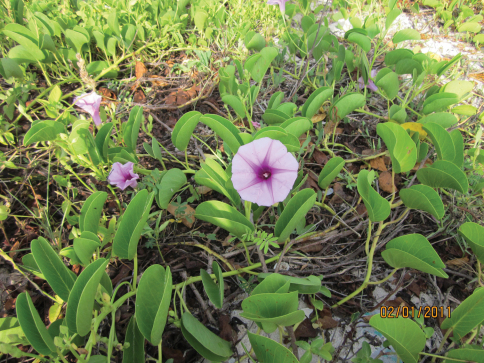
The host plant *Ipomoea pescapre* (Convolvulaceae) on the sea coast in Kikambala.

**Figure 2.**
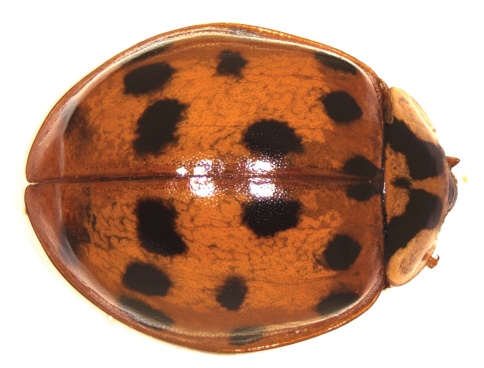
Female of *Harmonia axyridis* from Kikambala, colour morph *succinea*, with 19 spots and elytral ridge.

We observed the beetles on the plant *Ipomoea pescapre* (Convolvulaceae) on the sea coast (Fig. 1). All individuals belonged to the colour morph *succinea* (Hope), with 19 well-developed spots on the elytra and well-developed elytral ridges (Fig. 2). Like a previous record of *Harmonia axyridis* in Uruguay (Nedvěd and Krejčík 2010), this finding was done by chance by a non-professional entomologist.

## Discussion

Because Kikambala is a holiday centre, but not a port or transport node, we consider the occurrence of *Harmonia axyridis* here to be the result of a wider and lasting invasion, rather than a singular incidental and ephemeral introduction with goods.

The observed colour morph *succinea* is the most common morph in the eastern part of its natural range (Blekhman et al. 2010) and in the invasive European population (Brown et al. 2008). The size of the spots suggests that the individuals recorded emerged from pupae at temperatures of around 25°C – the spots would be smaller or missing at higher temperatures (Michie et al. 2010).

High temperature may be limiting the continued spread of *Harmonia axyridis*, at least at a local scale. The American (Acar et al. 2001) and European (Fois et al. unpublished) invasive populations do not survive temperatures above 33°C. However, the CLIMEX model that used known physiological limits of *Harmonia axyridis* indicated that this species may tolerate most southern and eastern African countries, including Kenya (Poutsma et al. 2008). The coastal climate near Mombasa is rather hot (average annual temperature 26°C, Climate & Temperature 2011), while at higher elevations inland, mild temperatures (*e.g.* 18°C in the capital, Nairobi) are more favourable for *Harmonia axyridis*.

Although there were several independent introductions of *Harmonia axyridis* in Europe and North America, with different source populations from East Asia, there is a single main invasive population/strain in several continents (Lombaert et al. 2010). Thus in future the origin of the population in Kenya should be compared with known populations from both the native and invasive ranges, using molecular genetic methods (Blekhman et al. 2010, Thomas et al. 2010, Lawson Handley et al. in press) to determine if it is the same strain, or a different one that might have higher temperature requirements.

## Conclusion

We consider that *Harmonia axyridis* has established in Kenya, the first fully tropical country to be invaded, but that its further spread may be hampered by high temperature and low prey availability. In this region we suggest that *Harmonia axyridis* may pose a low threat to biodiversity, such as the native ladybird beetles, which are mostly coccidophagous.
